# Changes in Metabolic Profiles of Pea (*Pisum sativum* L.) as a Result of Repeated Short-Term Soil Drought and Subsequent Re-Watering

**DOI:** 10.3390/ijms23031704

**Published:** 2022-02-01

**Authors:** Lesław Bernard Lahuta, Joanna Szablińska-Piernik, Marcin Horbowicz

**Affiliations:** Department of Plant Physiology, Genetics and Biotechnology, University of Warmia and Mazury in Olsztyn, Oczapowskiego Street 1A/103A, 10719 Olsztyn, Poland; joanna.szablinska@uwm.edu.pl (J.S.-P.); marcin.horbowicz@uwm.edu.pl (M.H.)

**Keywords:** *Pisum sativum*, drought, metabolite profiling, proline, GABA, *myo*-inositol, raffinose

## Abstract

The metabolic re-arrangements of peas (*Pisum sativum* L.) under soil drought and re-watering are still not fully explained. The search for metabolic markers of the stress response is important in breeding programs, to allow for the selection drought-resistant cultivars. During the present study, changes in the polar metabolite content in pea plant shoots were measured under repeated short-term soil drought and subsequent re-watering. A gas chromatograph, equipped with a mass spectrometer (GC-MS), was used for the metabolite profiling of pea plants during their middle stage of vegetation (14–34 days after sowing, DAS). The major changes occurred in the concentration of amino acids and some soluble carbohydrates. Among them, proline, γ-aminobutyric acid (GABA), branched-chain amino acids, hydroxyproline, serine, *myo*-inositol, and raffinose were accumulated under each soil drought and decreased after re-watering. Besides, the obtained results show that the first drought/re-watering cycle increased the ability of pea plants to restore a metabolic profile similar to the control after the second similar stress. The accumulation of proline seems to be an important part of drought memory in pea plants. However, confirmation of this suggestion requires metabolite profiling studies on a broader spectrum of pea cultivars.

## 1. Introduction

The predicted further global temperature increase in the 21st century, together with the disruption of water retention in soils, increases the risk of both periodic droughts and the successive desertification of crop fields [1,2,3]. Therefore, to meet the increasing demand for food, feed and plant raw materials for industry (e.g., in the paper, chemical, pharmaceutical, energy, construction industries), it has become necessary to intensify research, to understand more completely the response of crop plants to soil drought stress [4,5]. Cultivated plants are exposed to short or long-term water stress at various stages of their development. Water stress is caused by reduced soil moisture due to rainfall deficit, transpiration and evaporation from the soil surface, and reduced air relative humidity. Water stress occurs especially in the absence of precipitation and increased temperature, as well as stronger winds, which increases evaporation [6]. This leads to a deficit between water uptake by the roots and water loss by the aboveground parts of the plant. Typical symptoms of water stress are usually inhibition of growth, loss of turgor, and wilting of plant organs, accompanied by a decrease in photosynthesis and changes in carbon and nitrogen metabolism [6,7]. The first defensive reactions of plants to water deficit are stomatal closure and reduced transpiration, as well as increased accumulation of osmotically active inorganic ions and other metabolites in root and leaf cells [8]. As a result, the osmotic potential of the cells increases, which inhibits further water loss and allows the roots to continue the uptake of water from the soil. This osmotic adjustment is the primary defense mechanism of plants in response to water deficit [9,10]. The ability of a plant to survive a long-term water deficit depends on the synthesis and accumulation of osmolytes [11]. These compounds are well soluble in water, small in size, lack surface charge and have low molecular polarity and act as osmoprotectants, stabilizing protein conformation, membranes and redox balance [12]. These include the following: (i) low molecular weight non-reducing sugars (sucrose, galactinol, raffinose, trehalose, fructans) [13,14,15,16]; (ii) polyols (mannitol, sorbitol, *myo*-inositol and its methyl ether derivatives) [17,18,19,20]; (iii) amino acids (proline and γ-aminobutyric acid (GABA)) [21,22]; (iv) other amino compounds (such as betaines and polyamines) [23,24]. The accumulation of some of these compounds is a species-specific feature but can also be the result of stress, as has been shown in metabolomics studies of crops, such as maize and soybean [25,26].

The accumulation of protective compounds in plants under stress is associated with changes in the metabolome, transcriptome, and proteome, reflecting a global reprogramming of metabolic pathways [20,27,28,29]. The biggest changes concern the primary metabolism, which results in the majority of the differences in the content of polar metabolites [20,27]. Although changes in metabolite content, in response to abiotic stresses, such as drought, salinity, and heat have been reviewed [20,30,31,32], much less is known about changes in the metabolome of plants exposed to repeated stresses [33,34,35,36]. It is supposed that metabolic changes in the first stress can be well established and activated in the next stress episode [37,38]. Our previous studies showed that under long-term soil drought conditions, there was a uniformly increased accumulation of proline, malate, and *myo*-inositol in all parts of the shoot in semi-leafed pea cultivars, and during plant re-watering, their concentration decreased [39]. This may confirm the critical role of these osmolytes under drought stress [8,9,40,41]. However, there is still a lack of data on the rearrangement of the pea metabolome under repeated drought stress. Therefore, a comparative study of polar metabolic profiles in pea shoots, in plants subjected to repeated cycles of short-term soil drought and re-watering, was undertaken. Since the general physiological responses of pea to drought are already known [40,41,42,43], the metabolomic studies undertaken involved plants exposed to soil drought until visible wilting and then after full turgor was restored. The gas chromatography-mass spectrometry (GC-MS) method, which is often used in the study of primary metabolism [20,27,39,44], has been applied for the qualitative and quantitative analysis of polar metabolites. The major objective of this study was to search for a compound, or compounds, that could be considered the metabolic marker(s) of pea response to drought, which could be important in breeding programs [44,45].

## 2. Results and Discussion

### 2.1. The Effect of Short-Term Soil Drought on Plant Growth and Development

**Figure 1:**
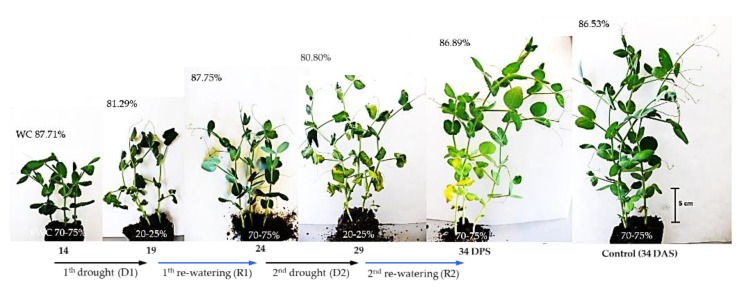
Plants of pea (*Pisum sativum* L., cv. Hubal) during repeated short-term soil drought during 14–19 DAS and 24–29 DAS, and subsequent re-watering (19–24 and 29–34 DAS). Control plants at 34 DAS are shown on the right. The values in the top left-hand corner of each picture indicate the water content (WC) in the shoot, whereas the bottom indicates soil water capacity (in %).

**Figure 2:**
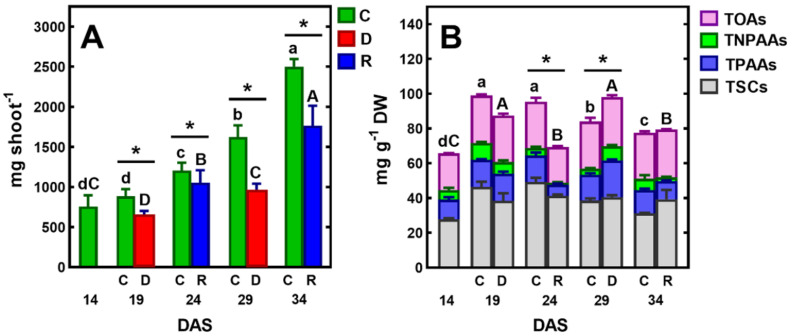
Changes in the fresh weight (FW) of the pea shoot (**A**) and the concentration of total identified polar metabolites (TIPMs), including major fractions (**B**): soluble carbohydrates (TSCs), protein amino acids (TPAAs), non-protein amino acids (TNPAAs) and organic acids (TOAs) during plant’s vegetation at control conditions (C) or exposed to short-term soil droughts (D) and re-watering (R). Values are means (*n* = 3) ± SD. The same letters above the bars indicate no statistically significant differences (*p* < 0.05), based on ANOVA and the Tukey post-hoc test for control plants (a–d, 14–34 DAS) or plants before and after soil drought and followed by recovery (A–C). The significant differences between TIPMs in shoots after 5 days of drought or re-watering and TIPMs in control plants (from the same DAS) were marked with an asterisk, based on the Student *t*-test (*, *p* < 0.05).

The effect of a drought on the physiological and biochemical processes in plants depends on its intensity, timing, and duration [41]. In plants, one of the main strategies for surviving unfavorable environmental conditions, including drought, is to limit the growth of aboveground parts [6]. In the present study, between 14 and 34 days after sowing (DAS) at optimum soil moisture, dynamic changes in the growth and shoot development of pea plants were observed. During this time, the fresh weight (FW) of the plants increased more than threefold, and three new nodes with leaves developed, while the shoot reached a height of about 20–25 cm at 34 DAS (Figure 1 and Figure 2). During this period, the water content (WC) of plant tissues remained almost constant, at 86–87%. The first 5-day irrigation interruption period, conducted from 14 to 19 DAS, reduced WC in shoots to 81.29% and resulted in an inhibition of shoot growth and shoot wilting (Figure 1).

After five days of re-watering, the shoots had recovered their growth and their FW increased (Figure 2A). Similar changes occurred after applying another cycle of soil drought and re-watering, which resulted in the restoration of plant turgor, growth, and the emergence of new leaves. However, in this case, one–two older leaves turned yellow and gradually dried.

Although plants survived both short-term drought/re-watering cycles (Figure 1), stress decreased the accumulation of FW (Figure 2A) and DW, which increased by ca 155 mg, while in control plants this was 260 mg. The results of growth inhibition and biomass accumulation, obtained in the present study, confirm those previously published on the effects of soil drought on pea plants [40,41,42,43]. Water stress inhibited vegetative growth, biomass accumulation, water content and photosynthetic pigments of the pea cultivars evaluated [42]. In this laboratory experiment, sensitivity to water stress was cultivar dependent. Field trials of peas also showed cultivar differences in response to soil water deficit [43]. Furthermore, the authors of this study showed that there is a decrease in chlorophyll content and leaf greenness in peas, in response to drought stress.

### 2.2. Metabolites in Shoots of Control Plants

Gas chromatography coupled to a quadrupole mass spectrometer (GC-MS) was used for the analysis of polar metabolites. This analytical technique allows for the identification and measurement of the content of a wide range of metabolites in biological tissues [39]. In pea tissues, we have identified many of these metabolites, including ten soluble carbohydrates, fifteen protein and three non-protein amino acids, eight organic acids, as well as phosphoric acid and urea (Table 1). The same polar metabolites have previously been found in various plant organs of semi-leafless peas [39]. In control plants, the concentration of total identified polar metabolites (TIPMs) increased between 14 and 19 DAS, from 72.97 to 104.77 mg/g DW, and then gradually decreased, until 34 DAS to 80.66 mg/g DW (Figure 2B).

Regardless of the plant developmental stage, total soluble carbohydrates (TSCs) shared 38–49% of TIPMs, whereas total organic acids (TOAs) ca 29%, and total protein and non-protein amino acids (TPAAs and TNPAAs, respectively) shared ca 22% of TIPMs. The major carbohydrates were sucrose, glucose and *myo*-inositol, occurring in the range 11.10–32.14, 3.75–8.58 and 3.85–7.29 mg/g DW, respectively. Among the organic acids, malic acid and citric acid were quantitatively the most dominant (16.13–22.64 and 3.50–4.07 mg/g DW, respectively), whereas among amino acids, asparagine (2.59–7.21 mg/g DW) and homoserine (2.46–6.24 mg/g DW). Moreover, significant contents of phosphoric acid were found (2.70–6.69 m/g DW, Table 1). Similar profiles and concentrations of polar metabolites occurred in tissues of semi-leafless pea cultivars during the vegetative stage of plant development, as was shown in our previous study [39]. Similarly, during the drought stress of soybean plants, most of the metabolites significantly increased in abundance or remained the same as in the control plants [26]. The presence of relatively high concentrations of sucrose, asparagine, and homoserine confirms the significant role of these metabolites as the major forms of carbon and nitrogen distributed in pea plants, from source to sink tissues [46,47]. The amino acids are loaded into phloem for partitioning to developing leaves, flowers, pods and seeds [46]. Amino acid transport and sucrose transport are crucial for seed development and quality [47].

**Figure 3:**
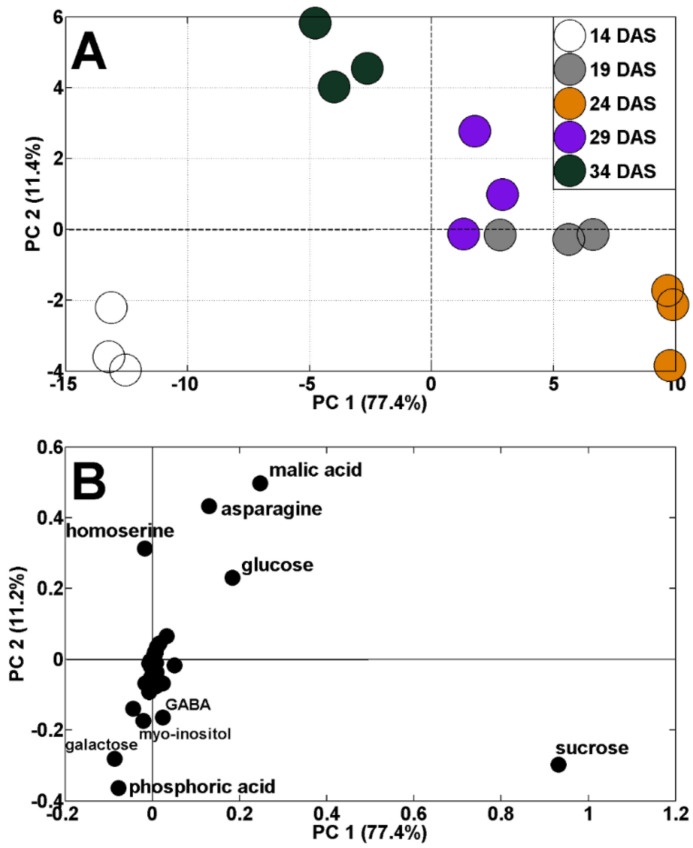
PCA of metabolite profiles of pea shoots during plant’s vegetation (14–34 DAS) at control conditions (**A**) and PCA loadings plots of polar metabolites (**B**).

Changes in TIPMs were accompanied by changes in metabolic profiles. The results of the principal components analysis (PCA) indicated a clear shift in the distribution of samples throughout vegetation. The results of samples collected at 14 and 34 DAS are located on the left, whereas results of samples from 19–29 DAS are on the right, from PC1 (sharing 77.4% of variability, Figure 3A). Moreover, the results of samples from 14 DAS were separated from those of 34 DAS, according to PC2 (11.4% of variability). The dispersion of the results of analyzed samples against PC1 was mainly determined by changes in sucrose, malic acid, asparagine, glucose, homoserine, galactose, and phosphoric acid concentrations (Figure 3B). The discrimination of samples during plant’s vegetation, by changes in primary metabolites, was documented in our earlier study in semi-leafless peas [39], as well as in other legumes [48,49,50].

### 2.3. Changes in Metabolic Profiles under Drought/Re-Watering Cycles

The short-term soil drought/re-watering cycles led to fluctuations in the concentration of TIPMs; its level significantly (*p* < 0.05) increased after droughts and decreased after recovery (Figure 2B). This was a result of changes in the concentrations of TPAAs and TNPAAs, while trends in changes of TSCs, TOAs and total remaining compounds (TRCs) were generally like those in control plants (Table 1). The increase in amino acid content confirms previous reports on the effect of drought stress on the amino acid accumulation in pea leaves and roots [39,51,52]. Similar effects were found in the leaves of soybean [26,53] and wheat [33,54,55]. Thus, the nitrogen metabolism seems to be susceptible to the disruption of water homeostasis under drought conditions.

**Figure 4:**
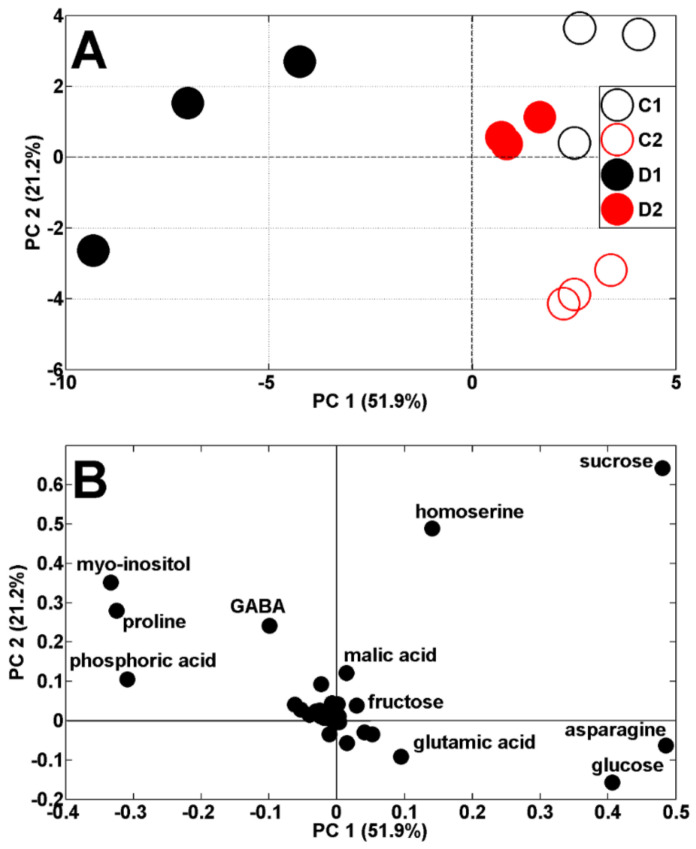
PCA of metabolite profiles of shoots of control (C1 and C2) and droughted (D1, D2) plants (samples collected at 19 and 29 DAS) (**A**). PCA loadings plots of polar metabolites are shown on (**B**).

Both drought periods affected the metabolic profiles of the pea shoots (Figure 4A). According to PCA score plots, the results of pea analysis after the first period of soil drought (19 DAS) are located on the left, while control results are on the right, against PC1 (51.9% of variability). The results after the second period of drought and control (29 DAS) are both located on the right, against PC1, and also separated from PC2 (21.2% of variability). The scattering of results was for the same metabolites as in the control plants (Figure 3B) as well as proline and *myo*-inositol (Figure 4B). It is interesting that *Medicago truncatula,* under drought stress conditions for proline and myo-inositol, showed similar regulatory profiles, indicating their contribution to drought tolerance in this species [29].

#### 2.3.1. Amino Acids

**Figure 5:**
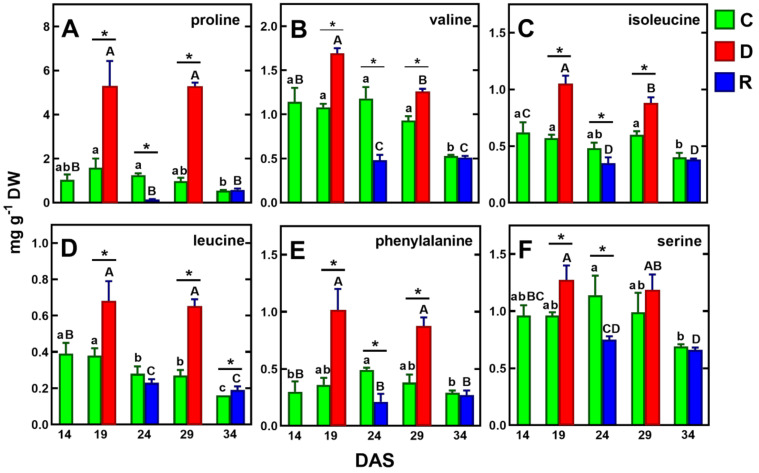
Changes in the concentration of selected amino acids, whose concentration increased under drought and decreased after re-watering (in both cycles) in pea shoots: proline (**A**), valine (**B**), isoleucine (**C**), leucine (**D**) phenylalanine (**E**) and serine (**F**). Values are means (*n* = 3) ± SD. The same letters above the bars indicate no statistically significant differences (*p* < 0.05), based on ANOVA and Tukey’s post-hoc test for control plants (a–d, 14–34 DAS) or plants before and after soil drought and followed by recovery (A–C). The significant differences between metabolite concentration in shoots after 5 days of drought (D) or re-watering (R) and metabolite in control (C) plants (from the same DAS) were marked with an asterisk, based on the Student *t*-test (*, *p* < 0.05).

The drought-induced proline accumulation in pea plants found in our study (Figure 5A) confirms previously published data [39,40,51]. The participation of proline in the plant’s responses to various stresses is well known [21]. The accumulation of this amino acid, in response to drought stress, was demonstrated in wheat [55], *Arabidopsis thaliana* [56], *Medicago truncatula* [29], white clover [57], and transgenic soybean [58]. Proline is considered an important osmotic regulatory component, playing a key role in mitigating both oxidative damage and stabilizing cell membranes [11,21]. The increased rate of proline biosynthesis in chloroplasts enables the maintenance of an adequate electron flow between photosystems and protects the photosynthetic apparatus from photoinhibition [59]. In turn, during salt stress, proline stabilizes cell respiration by protecting the mitochondrial electron chain [60]. Furthermore, the synthesis and accumulation of proline in the cytoplasm also indicates its osmoprotective properties [11].

In plants, proline accounts for about 5% of all free amino acids under favorable growth conditions, while under drought conditions, its share of amino acids can increase by up to 80% [59]. It was shown that under drought conditions, proline levels were dependent on stress duration and intensity and, under severe osmotic stress, proline content increased up to 100-fold compared to the control [60]. Indeed, in our study, the concentration of proline increased about fivefold after the first five-day drought and about 50-fold after the second drought, compared to its reduced levels after re-watering after the first drought (Figure 5A).

Proline contents were then 34 and 25% of all protein amino acids, respectively (Table 1). Interestingly, the concentration of proline after both drought periods was the same—5.26 mg/g DW (Table 1). Its content was similar to that in the leaves and roots of white clover subjected to drought for 7 days [57], but twofold higher than in the shoots of semi-leafless peas subjected to a mild 18-day drought [39]. These differences may be due to the increased transport of proline to the roots via phloem, as a result of increased water deficit [57]. During re-watering, proline concentration decreases significantly, which was confirmed in our study [61] (Figure 5A).

Similar to proline, after both periods of drought, pea plant tissues increased the concentrations of non-protein amino acid—γ-aminobutyric acid (GABA). After the first and second drought periods, its content increased from 1.92 to 4.14 mg/g DW and from 0.94 to 2.57 mg/g DW, respectively (Table 1). These results confirm previously published data regarding GABA accumulation in pea seedlings under drought conditions [62]. The present study also showed that the increase in the GABA level after the first period of drought was accompanied by a decrease in the level of its precursors, which are 2-keto-d-glutaric acid and glutamic acid (Table 1). GABA is synthesized by glutamate conversion, induced by stress conditions, which, in effect, can provide sufficient carbon/nitrogen for the TCA cycle and amino acid synthesis under drought conditions [62,63,64]. This in turn stimulates the accumulation of amino acids and organic acids which are involved in osmotic regulation [65]. Moreover, GABA is also considered to have a signaling role in both abiotic and biotic stresses [63,66].

Under drought/re-watering cycles, similar accumulation tendencies to proline and GABA were found for the branched-chain amino acids (BCAA: valine, isoleucine, leucine), phenylalanine and serine (Figure 5B–F). Although BCAA accumulation appears to be related to drought intensity, as demonstrated in wheat leaves [33], their role as osmolytes has not been confirmed to date [64]. The more than twofold increase in phenylalanine (Figure 4E) is likely related to the increased biosynthesis of secondary metabolites [65,67]. The increase in serine (Figure 5F), on the other hand, appears to be related to increased photorespiration [68,69]. It should be noted, however, that the above amino acids were accumulated at levels more than twofold lower than proline (Figure 5).

Concentrations of homoserine and asparagine, which were found to be the major amino acids in tissues of the control plants, decreased during the first drought/re-watering cycle, while they strongly increased (about 28- and 7-fold, respectively) in response to the second drought period, to 5.63 and 7.40 mg/g DW, and decreased during re-watering (Table 1). The accumulation of homoserine, but not asparagine, in pea leaves under drought has been shown previously [39,51]. It is possible that the drought-induced changes in homoserine, asparagine, and glutamine content were due to a disruption of phloem transport [52,70].

#### 2.3.2. Soluble Carbohydrates

**Figure 6:**
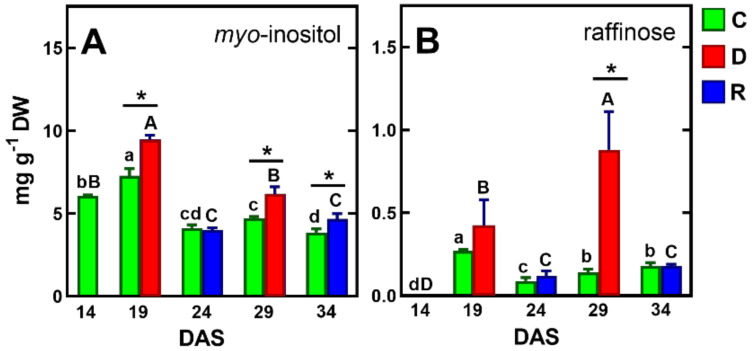
Changes in the concentration of *myo*-inositol (**A**) and raffinose (**B**) in control plants and plants exposed to drought and re-watering. The same letters above the bars indicate no statistically significant differences (*p* < 0.05), based on ANOVA and Tukey’s post-hoc test for control plants (a–d, 14–34 DAS) or plants before and after soil drought and followed by recovery (A–D). The significant differences between metabolite concentration in shoots after 5 days of drought (D) or re-watering (R) and metabolite in control (C) plants (from the same DAS) were marked with an asterisk, based on the Student t-test (*, *p* < 0.05).

The results of previous studies on the response of pea seedlings to soil drought [71] and osmotic stress [72], as well as mature pea plants to soil drought [39], showed a significant accumulation of sucrose in the tissues of this species. However, in the present study, under drought/re-watering conditions, sucrose contents increased similarly to control plants, up to 24 DAS, and then decreased slightly, remaining the major sugar (20–28 mg/g DW, Table 1). The contents of another important carbohydrate, glucose, decreased after drought and increased after re-watering (Table 1), In contrast, *myo*-inositol levels were elevated by drought and then decreased after re-watering, to levels similar to the control, and its accumulation of the first drought period was much higher than after the second (Figure 6A).

*Myo*-inositol is considered to play an important role in protecting tissues from oxidative damage by acting as an oxygen-free radical scavenger [73]. Moreover, it is a precursor in the synthesis of another important antioxidant, such as in ascorbic acid [74]. However, the antioxidative properties of *myo*-inositol seem to be questionable, although its synergistic action with other antioxidants, like glutathione or phenolic compounds, is possible [75]. It has also been shown that *myo*-inositol can act as an osmolyte as effectively as proline to prevent water loss and maintain cell turgor, as found in leaves of over-dried peppers (*Capsicum annuum*) [76].

Previous studies indicated an increase in raffinose content in pea response to drought stress, regardless of plant developmental stage, by which raffinose was considered a marker of resistance to this stress [39,71,72,77,78].

Similar results were obtained in the present study. However, the concentration of raffinose was below 1 mg/g DW, which may partly challenge the hypothesis of its protective properties [14,77]. It is perhaps crucial for the protective function of raffinose is its intracellular localization in the cytoplasm or chloroplasts [78].

#### 2.3.3. Organic Acids and Other Compounds

Organic acid contents were generally maintained at the same level in both control and plants exposed to drought cycles (Table 1). The exception was malic acid, which was significantly reduced after the first drought period. Phosphoric acid concentration decreased during plant vegetation, in both control plants and plants subjected to drought-re-watering cycles, and was 6.35–2.60 and 6.79–3.14 mg/g DW, respectively (Table 1). The small fluctuations in urea content indicate that the urea cycle is maintained under stress conditions, although drought may affect this pathway, as was shown in drought-treated maize grains [79] and artichoke seedlings [80].

### 2.4. Metabolic Profiles of Re-Watered Pea Plants

**Figure 7:**
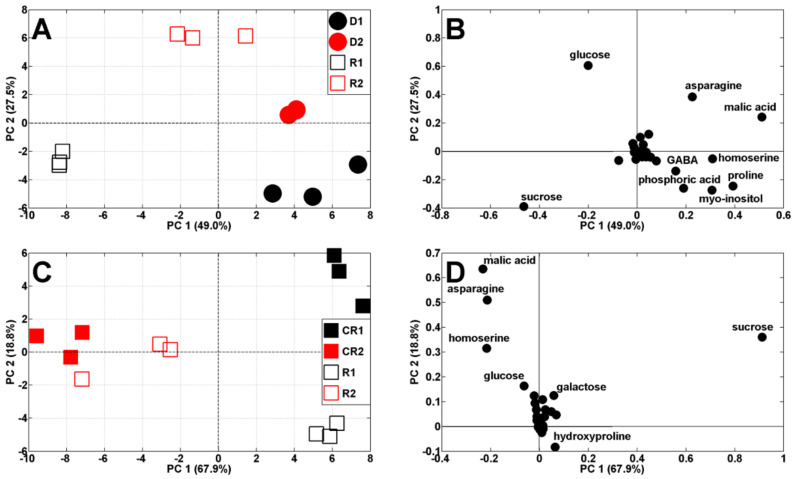
PCA of metabolite profiles of: (**A**) droughted (D1 and D2, 14–19 and 24–29 DAS) and re-watered (R1 and R2, 19–24 and 29–34 DAS) and (**C**) re-watered (R1 and R2) and appropriate control plants (CR1 and CR2, at 24 and 34 DAS, respectively). Appropriate PCA loading plots of polar metabolites are shown in (**B**,**D**).

After re-watering, the metabolic profiles of droughted plants and re-watered plants changed again and they were separated according to PC1 (49% of variability) (Figure 7A). Moreover, results from samples after the first drought/re-watering cycle are at the bottom, while for after the second cycle, they are at the top, against PC2 (27.5%). The comparison of twice droughted/re-watered plants, with appropriate controls, revealed that the separation between samples decreased after the second drought/re-watering cycle (Figure 7C).

The level of TIPMs in pea plants after the second drought/re-watering cycle was similar to the control plants (82.75 and 80.66 mg/g DW, Figure 7B, Table 1), whereas after the first cycle, it was about 27% lower than in the control, mainly due to lower contents of amino acids and malate (Table 1). However, after re-watering, TSCs accounted for 57 and 47% of TIPMs after the first and second drought/re-watering cycle, respectively, which was 10% higher than in the control (Table 1).

Under field conditions, plants are often exposed to repeated episodes of drought and watering. Metabolic, transcriptomic, and proteomic responses of the plant to repeated drought/re-watering cycles appear to play a key role in effective recovery from this stress [35]. Therefore, researchers have attempted for many years to approximate and elucidate the responses of plant species to this common and complex stress [34,36,37,81,82,83]. The results of these studies showed that plants can strengthen their defenses by retaining information from previous stress periods [35]. So-called drought stress memory in plants includes processes related to photosynthesis, respiration, osmotic regulation, their protective functions, and maintenance of water status [81,83]

Our study confirms that after returning from the second drought period, the contents of each metabolite group were higher compared to control plants. Thus, it seems that the first drought/re-watering cycle increased the ability of plants to restore a metabolic profile similar to the control, after the second similar stress. The current study shows that proline accumulation appears to be an important part of drought memory in pea plants. Drought/re-watering cycles had less effect on γ-aminobutyric acid (GABA) and branched-chain amino acids, and on *myo*-inositol and raffinose

The legumes grow in symbiosis with the *Rhizobium* bacteria, and soil drought affects these organisms as well [82]. They are responsible for nitrogen metabolism in plants. This additional factor makes studies on the effects of drought stress in legumes more difficult to evaluate than those plants that do not grow in symbiosis with *Rhizobium*.

## 3. Materials and Methods

### 3.1. Material

Seeds of pea (*Pisum sativum* L. cv. Hubal), a conventionally leaved variety, were purchased from a domestic seed company (DANKO Plant Breeding, Choryń, Poland). Seeds were surface decontaminated (1 min, 60% ethanol), rinsed several times with double distilled water, surface dried on filter paper and sown in plastic seedling trays (32 × 32 × 5 cm), three seeds each of 25 cells, filled with 70 cm^3^ of garden soil—Substral Osmocote (Garden e-Commerce, Reda, Poland). The soil moisture was kept at 70–75% field water capacity (FWC), measured using a soil moisture meter (ThetaProbe ML3, Delta-T Devices Ltd., Cambridge, UK). Plants were cultivated in the spring of 2017, from mid-April to mid-May, in a greenhouse laboratory at the University of Warmia and Mazury in Olsztyn (20°30′ E 53°47′ N). The day and night temperatures ranged from 22 to 30 °C and from 16 to 20 °C in April and May, respectively.

Plants were exposed to soil drought by cessation of plant watering from the 14th day after sowing (DAS), when plants reached stage V3 (the third true leaf with one pair of leaflets had unfolded at the third node, tendril was present). After five days, the fresh weight content (FWC) decreased to 20–25% and the first symptoms of plant wilting appeared (Figure 1). Then, watering was resumed for 5 days, enabling the recovery of full turgor and plant growth. The watering cessation was by re-watering (for 5 days each period) was repeated. Control plants grew at optimal FWC (70–75%) until 34 DAS.

### 3.2. Methods

Shoots from control and twofold droughted and re-watered plants, collected at 14, 19, 24, 29 and 34 DAS (in 3 replicates at each harvest time), were weighed and frozen in liquid nitrogen. The time elapsing between the sample collection and their freezing was no longer than 10 min. Samples were stored in an ultra-refrigerator (for 7 days at −76 °C) and freeze-dried for 48 h (shelf freeze-dryer, Alpha 1–2 LD, Martin Christ, Osterode am Harz, Germany). The water concentration (WC) was calculated as the difference between the shoot’s fresh weight (FW) and dry weight (DW) and expressed as a percentage of FW.

#### 3.2.1. Analysis of Polar Metabolites

The extraction of polar metabolites was carried out according to the method described earlier [39]. Polar metabolites were extracted from 40 to 42 mg of dry tissues with 900 μL of the mixture of methanol, as follows: water (1:1, *v*/*v*, containing 100 μg of ribitol as internal standard) at 70 °C for 30 min. The extraction was repeated four times, and the obtained crude extracts were collected and centrifuged (20,000× *g* at 4 °C for 20 min). Then portions of the supernatant (600 μL) were mixed with 400 μL of cold chloroform to remove nonpolar compounds. After centrifugation, the polar fraction was evaporated to dryness in a speed vacuum rotary evaporator (JW Electronic, Warsaw, Poland). The derivatization of the metabolites involved the use of 40 μL of *O*-methoxamine hydrochloride (at a concentration of 20 mg mL^−1^ of pyridine), heating at 37 °C for 75 min (with continuous shaking, 500 rpm) and an additional 160 μL of the mixture of MSTFA (*N*-methyl-*N*-trimethylsilyl-trifluoroacetamide) with pyridine (1:1, *v/v* ratio, at 70 °C for 30 min), according to Lisec et al. [84]. The mixtures of TMS derivatives were separated on a ZEBRON ZB-5MSi GUARDIAN capillary column (Phenomenex, Torrance, CA, USA) in a gas-chromatograph coupled with a quadrupole mass spectrometer (QP-GC-2010, Shimadzu, Kyoto, Japan). Metabolites were identified by comparison of retention time (RT), relative retention time (RRT), retention indices (RI), determined according to the saturated hydrocarbons and mass spectra of original standards derived from Sigma-Aldrich (Saint Louis, MO, USA) and from the NIST 05 library (National Institute of Standards and Technology, Gaithersburg, MD, USA). The concentration of identified polar metabolites was calculated according to the method described previously [39].

#### 3.2.2. Statistics

The results were subjected to one-way ANOVA with a post-hoc test (Tuckey) or Student’s *t*-test using Statistica software (version 12.0; StatSoft, Tulsa, OK, USA). Graphs were prepared using GraphPad Prism (version 3.0; GraphPad Software, San Diego, CA, USA). Principal Component Analysis (PCA) was performed in the COVAIN program [85], using the MATLAB software (version 2013a, Math Works, Natick, MA, USA), to compare the metabolic profiles of peas during vegetative growth as well as under soil drought/recovery cycles.

## 4. Conclusions

The exposure of peas (in the phase of leaf development and shoot growth) to two sequential short cycles of soil drought/re-watering, revealed similar changes in the metabolic profile of shoots during stress and recovery. The slowing down of plant growth during each drought period was associated with the rearrangement of the amino acid profile and, to a lesser extent, the soluble carbohydrates. The similarity in the accumulation of some metabolites (proline, GABA, *myo*-inositol and raffinose) during stress and a dramatic decrease in their content during re-watering confirm the participation of these compounds in the pea’s defense reaction to disturbances in water management. However, it seems that this defense may largely depend on the adaptation of the root system (including symbiosis with *rhizobia*) to stress conditions. Therefore, it is necessary to study the metabolic profiles of roots that can reveal their adaptation to soil drought. Moreover, it remains an open question whether the metabolic response is primed and increases the plants’ tolerance to severe drought.

## Figures and Tables

**Table 1:** The concentration of polar metabolites (in mg/g DW) in pea shoots (*Pisum sativum* L., cv. Hubal) during 20 days of plant vegetation (from the 14th to the 34th day after sowing, DAS) at optimal soil moisture (control), after 5 days of watering withdrawal from 14th to 19th and from 24th to 29th DAS (drought, D1 and D2, respectively) and followed re-watering for 5 days, between 19–24 and 29–34 DAS (R1 and R2, respectively).

Metabolites	Control					Drought (D)/Recovery (R)			
DAS	14	19	24	29	34	D1	R1	D2	R2
**TIPMs**, including:	**72.97 ^dC^**	**104.77 ^a^**	**99.96 ^a^**	**88.15 ^b^**	**80.66 ^c^**	**95.12 ^A^**	72.99 ^B^*	102.19 ^A^*	**82.75 ^B^**
**TSCs**, including:	**27.86 ^cB^**	**46.45 ^a^**	**49.34 ^a^**	**38.54 ^b^**	**31.28 ^c^**	**38.43 ^A^**	41.29 ^A^*	**40.50 ^A^**	**39.26 ^A^**
fructose	0.13 ^bD^	1.39 ^a^	1.04 ^ab^	0.75 ^ab^	0.37 ^b^	0.78 ^BC^	0.20 ^C^*	1.04 ^AB^	1.74 ^A^*
galactose	4.19 ^aA^	1.71 ^b^	2.44 ^ab^	0.78 ^b^	1.30 ^b^	1.41 ^B^	1.37 ^B^	1.05 ^B^	0.80 ^B^*
glucose	3.75 ^bBC^	7.12 ^ab^	8.58 ^a^	7.89 ^a^	7.71 ^a^	3.44 ^C^*	6.53 ^B^	6.25 ^BC^*	10.85 ^A^
sucrose	11.10 ^eC^	26.98 ^b^	32.14 ^a^	23.48 ^c^	17.32 ^d^	21.34 ^B^	28.00 ^A^*	23.89 ^AB^	20.22 ^B^
galactinol	0.12 ^aB^	0.08 ^ab^	0.06 ^b^	0.05 ^b^	0.05 ^b^	0.11 ^B^*	0.04 ^B^	0.45 ^A^*	0.00 ^B^*
raffinose	0.00 ^dD^	0.27 ^a^	0.09 ^c^	0.14 ^b^	0.18 ^b^	0.42 ^B^	0.12 ^C^	0.88 ^A^*	0.18 ^C^
*myo*-inositol	6.08 ^bB^	7.29 ^a^	4.13 ^cd^	4.70 ^c^	3.85 ^d^	9.47 ^A^*	4.01 ^C^	6.19 ^B^*	4.67 ^C^*
mannitol	0.11 ^aA^	0.06 ^a^	0.07 ^a^	0.11 ^a^	0.04 ^a^	0.07 ^AB^	0.06 ^AB^	0.05 ^B^	0.04 ^B^
ribonic acid	0.46 ^aAB^	0.36 ^b^	0.31 ^bc^	0.32 ^bc^	0.24 ^c^	0.46 ^AB^*	0.53 ^A^*	0.36 ^B^	0.38 ^B^*
fructose-6-phosphate	1.91 ^aA^	1.19 ^b^	0.48 ^c^	0.31 ^d^	0.22 ^d^	0.94 ^B^*	0.42 ^C^	0.35 ^C^	0.40 ^C^*
**TPAAs**, including:	**11.36 ^bC^**	**15.67 ^a^**	**15.12 ^a^**	**14.86 ^a^**	**13.26 ^ab^**	**15.61 ^B^**	6.34 ^D^*	21.14 ^A^*	10.57 ^C^*
alanine	0.00 ^cB^	0.17 ^a^	0.00 ^c^	0.00 ^c^	0.08 ^b^	0.16 ^A^	0.00 ^B^	0.16 ^A^*	0.04 ^B^
asparagine	2.59 ^bBC^	6.85 ^a^	5.60 ^a^	6.23 ^a^	7.21 ^a^	1.56 ^C^*	1.11 ^C^*	7.40 ^A^	4.55 ^B^*
aspartic acid	0.81 ^aABC^	1.03 ^a^	1.28 ^a^	1.26 ^a^	1.22 ^a^	0.62 ^BC^*	0.45 ^C^*	1.14 ^A^	0.99 ^AB^
glutamic acid	0.46 ^bB^	0.89 ^ab^	1.30 ^ab^	1.47 ^a^	1.06 ^ab^	0.15 ^B^*	0.21 ^B^*	0.88 ^A^	1.14 ^A^
glutamine	0.29 ^aA^	0.13 ^c^	0.17 ^bc^	0.16 ^bc^	0.20 ^b^	0.16 ^B^	0.29 ^A^*	0.11 ^B^	0.17 ^B^
glycine	0.38 ^aAB^	0.33 ^b^	0.30 ^cd^	0.22 ^de^	0.18 ^e^	0.45 ^A^	0.18 ^B^	0.33 ^AB^*	0.21 ^AB^*
hydroxyproline	1.18 ^aAB^	0.54 ^bc^	0.95 ^ab^	0.97 ^ab^	0.31 ^c^	0.87 ^BC^	1.65 ^A^*	0.37 ^C^*	0.62 ^BC^*
isoleucine	0.62 ^aC^	0.57 ^a^	0.48 ^ab^	0.60 ^a^	0.40 ^b^	1.05 ^A^*	0.35 ^D^*	0.88 ^B^*	0.38 ^D^
leucine	0.39 ^aB^	0.38 ^a^	0.28 ^b^	0.27 ^b^	0.16 ^c^	0.68 ^A^*	0.23 ^C^	0.65 ^A^*	0.19 ^C^*
lysine	0.11 ^aB^	0.09 ^a^	0.11 ^a^	0.09 ^a^	0.05 ^a^	0.15 ^AB^	0.00 ^C^*	0.17 ^A^*	0.00 ^C^*
phenylalanine	0.30 ^bB^	0.36 ^ab^	0.49 ^a^	0.38 ^ab^	0.29 ^b^	1.01 ^A^*	0.21 ^B^*	0.86 ^A^*	0.27 ^B^
proline	1.04 ^abB^	1.58 ^a^	1.25 ^a^	0.98 ^ab^	0.55 ^b^	5.26 ^A^*	0.14 ^B^*	5.26 ^A^*	0.58 ^B^
serine	0.96 ^abBC^	0.96 ^ab^	1.14 ^a^	0.99 ^ab^	0.69 ^b^	1.27 ^A^*	0.75 ^CD^*	1.18 ^AB^	0.66 ^D^
threonine	1.10 ^aA^	0.70 ^b^	0.60 ^b^	0.34 ^c^	0.34 ^c^	0.53 ^B^*	0.29 ^C^*	0.49 ^B^*	0.27 ^C^
valine	1.14 ^aB^	1.08 ^a^	1.18 ^a^	0.93 ^a^	0.53 ^b^	1.69 ^A^*	0.48 ^C^*	1.26 ^B^*	0.51 ^C^
**TNAAs**, including:	**5.43 ^bB^**	**9.53 ^a^**	**4.37 ^b^**	**3.51 ^b^**	**6.46 ^ab^**	6.68 ^AB^*	1.14 ^C^*	8.23 ^A^*	2.09 ^C^*
β-alanine	0.09 ^aA^	0.13 ^a^	0.10 ^a^	0.00 ^b^	0.00 ^b^	0.03 ^B^*	0.00 ^B^*	0.02 ^B^*	0.03 ^B^
GABA	1.92 ^bAB^	3.16 ^a^	1.67 ^bc^	1.04 ^bc^	0.66 ^c^	3.14 ^A^	0.94 ^B^*	2.57 ^A^*	0.73 ^B^
homoserine	3.42 ^abAB^	6.24 ^a^	2.59 ^b^	2.46 ^b^	5.80 ^a^	3.51 ^AB^*	0.20 ^C^*	5.63 ^A^*	1.32 ^BC^*
**TOAs**, including:	**21.10 ^bB^**	**27.43 ^a^**	**26.64 ^a^**	**27.20 ^a^**	**26.50 ^a^**	**26.67 ^A^**	20.64 ^B^*	**28.14 ^A^**	**27.48 ^A^**
citric acid	4.07 ^aA^	4.05 ^a^	3.86 ^a^	3.88 ^a^	3.50 ^a^	3.46 ^A^	3.91 ^A^	3.97 ^A^	3.90 ^A^*
2-keto-d-glutaric acid	0.02 ^bC^	0.04 ^a^	0.03 ^a^	0.04 ^a^	0.02 ^b^	0.07 ^A^*	0.05 ^B^*	0.06 ^AB^*	0.00 ^D^*
fumaric acid	0.14 ^aA^	0.13 ^a^	0.15 ^a^	0.11 ^a^	0.16 ^a^	0.13 ^A^	0.13 ^A^	0.16 ^A^*	0.15 ^A^
malic acid	16.13 ^bB^	22.64 ^a^	21.43 ^a^	22.17 ^a^	21.75 ^a^	22.43 ^A^	15.68 ^B^*	23.24 ^A^	22.14 ^A^
oxalic acid	0.05 ^aA^	0.04 ^b^	0.05 ^a^	0.05 ^a^	0.03 ^b^	0.06 ^A^	0.04 ^A^	0.04 ^A^*	0.04 ^A^
lactic acid	0.10 ^aA^	0.08 ^a^	0.08 ^a^	0.11 ^a^	0.05 ^a^	0.07 ^A^	0.08 ^A^	0.08 ^A^	0.07 ^A^
propanoic acid	0.52 ^bB^	0.42 ^b^	1.03 ^a^	0.84 ^ab^	0.97 ^a^	0.45 ^B^	0.67 ^B^*	0.57 ^B^	1.11 ^A^
shikimic acid	0.03 ^aA^	0.04 ^a^	0.00 ^b^	0.00 ^b^	0.00 ^b^	0.00 ^C^*	0.06 ^A^*	0.03 ^B^*	0.07 ^A^*
**TRCs**, including:	**7.01 ^aA^**	**5.69 ^b^**	**4.49 ^c^**	**4.04 ^c^**	**3.17 ^d^**	**7.72 ^A^**	3.59 ^B^*	**4.18 ^B^**	**3.35 ^B^**
phosphoric acid	6.69 ^aA^	5.12 ^b^	4.10 ^c^	3.57 ^c^	2.70 ^d^	7.11 ^A^	3.42 ^B^	3.66 ^B^	3.06 ^B^
urea	0.31 ^aB^	0.58 ^a^	0.39 ^a^	0.47 ^a^	0.47 ^a^	0.61 ^A^	0.17 ^C^	0.51 ^AB^	0.28 ^B^

Abbreviations: DAS—day after sowing; TIPMs—total identified polar metabolites; TPAAS— total protein amino acids; TNAAs—total non-protein amino acids; TOAs—total organic acids; TRCs—total remaining compounds. The same superscript letters by the values indicate no statistically significant differences (*p* < 0.05) based on ANOVA analysis and Tukey’s post-hoc test for control plants (a–d) and before stress (14 DAS) and subjected to soil drought and followed re-watering (A–D) (valid in rows). The significant differences (Student’s *t*-test, *p* < 0.05) between plants subjected to soil drought (D1, D2) and control plants (at 19 and 29 DAS) or after recovery (R1, R2) and control plants (24 and 34 DAS) were marked with a superscript asterisk.

## Data Availability

The data presented in this study are available in this article.

## References

[1] Berg A., Sheffield J., Milly P.C.D. (2017). Divergent surface and total soil moisture projections under global warming. Geophys. Res. Lett..

[2] Pokhrel Y., Hanasaki N., Yeh P.F., Yamada T.Y., Kanae S., Oki T. (2012). Model estimates of sea-level change due to anthropogenic impacts on terrestrial water storage. Nat. Geosci..

[3] Kumar S., Lawrence D.M., Dirmeyer P.A., Sheffield J. (2014). Less reliable water availability in the 21st century climate projections. Earth’s Future.

[4] Raza A., Razzaq A., Mehmood S.S., Zou X., Zhang X., Lv Y., Xu J. (2019). Impact of climate change on crops adaptation and strategies to tackle its outcome: A review. Plants.

[5] Foley J.A., Ramankutty N., Brauman K.A., Cassidy E.S., Gerber J.S., Johnston M., Mueller N.D., O’Connell C., Ray D.K., West P.C. (2011). Solutions for a cultivated planet. Nature.

[6] Kooyers N.J. (2015). The evolution of drought escape and avoidance in natural herbaceous populations. Plant Sci..

[7] Baslam M., Mitsui T., Sueyoshi K., Ohyama T. (2021). Recent advances in carbon and nitrogen metabolism in C3 plants. Int. J. Mol. Sci..

[8] Suprasanna P., Nikalje G.C., Rai A.N., Iqbal N., Nazar R., Khan N.A. (2016). Osmolyte accumulation and implications in plant abiotic stress tolerance. Osmolytes and Plants Acclimation to Changing Environment: Emerging Omics Technologies.

[9] Blum A. (2017). Osmotic adjustment is a prime drought stress adaptive engine in support of plant production. Plant Cell Environ..

[10] Ozturk M., Turkyilmaz Unal B., García-Caparrós P., Khursheed A., Gul A., Hasanuzzaman M. (2021). Osmoregulation and its actions during the drought stress in plants. Physiol. Plant..

[11] Yancey P.H. (2005). Organic osmolytes as compatible, metabolic and counteracting cytoprotectants in high osmolarity and other stresses. J. Exp. Biol..

[12] Slama I., Abdelly C., Bouchereau A., Flowers T., Savouré A. (2015). Diversity, distribution and roles of osmoprotective compounds accumulated in halophytes under abiotic stress. Ann. Bot..

[13] Ruan Y.L. (2014). Sucrose metabolism: Gateway to diverse carbon use and sugar signaling. Annu. Rev. Plant Biol..

[14] Sengupta S., Mukherjee S., Basak P., Majumder A.L. (2015). Significance of galactinol and raffinose family oligosaccharide synthesis in plants. Front. Plant Sci..

[15] Iordachescu M., Imai R. (2008). Trehalose biosynthesis in response to abiotic stresses. J. Integr. Plant Biol..

[16] Valluru R., Van den Ende W. (2008). Plant fructans in stress environments: Emerging concepts and future prospects. J. Exp. Bot..

[17] Abebe T., Guenzi A.C., Martin B., Cushman J.C. (2013). Tolerance of mannitol-accumulating transgenic wheat to water stress and salinity. Plant Physiol..

[18] Wang Z., Stutte G.W. (1992). The role of carbohydrates in active osmotic adjustment in apple under water stress. J. Am. Soc. Hortic. Sci..

[19] Valluru R., Van den Ende W. (2011). *Myo*-inositol and beyond—Emerging networks under stress. Plant Sci..

[20] Krasensky J., Jonak C. (2012). Drought, salt, and temperature stress-induced metabolic rearrangements and regulatory networks. J. Exp. Bot..

[21] Szabados L., Savouré A. (2010). Proline: A multifunctional amino acid. Trends Plant Sci..

[22] Vijayakumari K., Jisha K.C., Puthur J.T. (2016). GABA/BABA priming: A means for enhancing abiotic stress tolerance potential of plants with less energy investments on defense cache. Acta Physiol. Plant..

[23] McNeil S.D., Nuccio M.L., Hanson A.D. (1999). Betaines and related osmoprotectants. Targets for metabolic engineering of stress resistance. Plant Physiol..

[24] Chen D., Shao Q., Yin L., Younis A., Zheng B. (2019). Polyamine function in plants: Metabolism, regulation on development, and roles in abiotic stress responses. Front. Plant Sci..

[25] Obata T., Witt S., Lisec J., Palacios-Rojas N., Florez-Sarasa I., Yousfi S., Araus J.L., Cairns J.E., Fernie A.R. (2015). Metabolite profiles of maize leaves in drought, heat, and combined stress field trials reveal the relationship between metabolism and grain yield. Plant Physiol..

[26] Das A., Rushton P.J., Rohila J.S. (2017). Metabolomic profiling of soybeans (*Glycine max* L.) reveals the importance of sugar and nitrogen metabolism under drought and heat stress. Plants.

[27] Sanchez D.H., Schwabe A.F., Erban B.A., Udvardi M.K., Kopka J. (2012). Comparative metabolomics of drought acclimation in model and forage legumes. Plant Cell Environ..

[28] Rodziewicz P., Swarcewicz B., Chmielewska K., Wojakowska A., Stobiecki M. (2014). Influence of abiotic stresses on plant proteome and metabolome changes. Acta Physiol. Plant..

[29] Hong J., Yang L., Zhang D., Shi J. (2016). Plant metabolomics: An indispensable system biology tool for plant science. Int. J. Mol. Sci..

[30] Zhang J.-Y., Cruz de Carvalho M.H., Torres-Jerez I., Kang Y., Allen S.N., Huhman D.V., Tang Y., Murray J., Sumner L.W., Udvardi M.K. (2014). Global reprogramming of transcription and metabolism in *Medicago truncatula* during progressive drought and after rewatering. Plant Cell Environ..

[31] Obata T., Fernie A.R. (2012). The use of metabolomics to dissect plant responses to abiotic stresses. Cell. Mol. Life Sci..

[32] Jorge T.F., Rodrigues J.A., Caldana C., Schmidt R., van Dongen J.T., Thomas-Oates J., António C. (2016). Mass spectrometry-based plant metabolomics: Metabolite responses to abiotic stress. Mass Spectrom. Rev..

[33] Bowne J.B., Erwin T.A., Juttner J., Schnurbusch T., Langridge P., Bacic A., Roessner U. (2012). Drought responses of leaf tissues from wheat cultivars of differing drought tolerance at the metabolite level. Mol. Plant.

[34] Crisp P.A., Ganguly D., Eichten S.R., Borevitz J.O., Pogson B.J. (2016). Reconsidering plant memory: Intersections between stress recovery, RNA turnover, and epigenetics. Sci. Adv..

[35] Menezes-Silva P.E., Sanglard L.M.V.P., Ávila R.T., Morais L.E., Martins S.C.V., Nobres P., Patreze C.M., Ferreira M.A., Araújo W.L., Fernie A.R. (2017). Photosynthetic and metabolic acclimation to repeated drought events play key roles in drought tolerance in coffee. J. Exp. Bot..

[36] Da Fonseca-Pereira P., Daloso D.M., Gago J., de Oliveira Silva F.M., Condori-Apfata J.A., Florez-Sarasa I., Tohge T., Reichheld J.-P., Nunes-Nesi A., Fernie A.R. (2019). The mitochondrial thioredoxin system contributes to the metabolic responses under drought episodes in *Arabidopsis*. Plant Cell Physiol..

[37] Schwachtje J., Whitcomb S.J., Firmino A.A.P., Zuther E., Hincha D.K., Kopka J. (2019). Induced, imprinted, and primed responses to changing environments: Does metabolism store and process information?. Front. Plant Sci..

[38] Lephatsi M.M., Meyer V., Piater L.A., Dubery I.A., Tugizimana F. (2021). Plant responses to abiotic stresses and Rhizobacterial biostimulants: Metabolomics and epigenetics perspectives. Metabolites.

[39] Szablińska-Piernik J., Lahuta L.B. (2021). Metabolite profiling of semi-leafless pea (*Pisum sativum* L.) under progressive soil drought and subsequent re-watering. J. Plant Physiol..

[40] Sánchez F.J., Manzanares M., de Andres E.F., Tenorio J.L., Ayerbe L. (1998). Turgor maintenance, osmotic adjustment and soluble sugar and proline accumulation in 49 pea cultivars in response to water stress. Field Crops Res..

[41] Embiale A., Hussein M., Husen A., Sahile S., Mohammed K. (2016). Differential sensitivity of *Pisum sativum* L. cultivars to water-deficit stress: Changes in growth, water status, chlorophyll fluorescence and gas exchange attributes. J. Agron..

[42] Nemeskéri E., Molnár K., Vígh R., Nagy J., Dobos A. (2015). Relationships between stomatal behaviour, spectral traits and water use and productivity of green peas (*Pisum sativum* L.) in dry seasons. Acta Physiol. Plant..

[43] Magyar-Tábori K., Mendler-Drienyovszki N., Dobránszki J. (2011). Models and tools for studying drought stress responses in peas. OMICS J. Integr. Biol..

[44] Fernie A.R., Schauer N. (2010). Metabolomics-assisted breeding: A viable option for crop improvement?. Trends Genet..

[45] Alseekh S., Bermudez L., de Haro L.A., Fernie A.R., Carrari F. (2018). Crop metabolomics: From diagnostics to assisted breeding. Metabolomics.

[46] Zhang L., Garneau M.G., Majumdar R., Grant J., Tegeder M. (2015). Improvement of pea biomass and seed productivity by simultaneous increase of phloem and embryo loading with amino acids. Plant J..

[47] Lu M.-Z., Snyder R., Grant J., Tegeder M. (2020). Manipulation of sucrose phloem and embryo loading affects pea leaf metabolism, carbon and nitrogen partitioning to sinks as well as seed storage pools. Plant J..

[48] Goufo P., Moutinho-Pereira J.M., Jorge T.F., Correia C.M., Oliveira M.R., Rosa E.A.S., António C., Trindade H. (2017). Cowpea (*Vigna unguiculata* L. Walp.) metabolomics: Osmoprotection as a physiological strategy for drought stress resistance and improved yield. Front. Plant Sci..

[49] Gutsch A., Hendrix S., Guerriero G., Renaut J., Lutts S., Alseekh S., Fernie A.R., Hausman J.-F., Vangronsveld J., Cuypers A. (2020). Long-term Cd exposure alters the metabolite profile in stem tissue of *Medicago sativa*. Cells.

[50] Khan N., Bano A., Rahman M.A., Rathinasabapathi B., Babar M.A. (2019). UPLC-HRMS-based untargeted metabolic profiling reveals changes in chickpea (*Cicer arietinum*) metabolome following long-term drought stress. Plant Cell Environ..

[51] Charlton A.J., Donarski J.A., Harrison M., Jones S.A., Godward J., Oehlschlager S., Arques J.L., Ambrose M., Chinoy C., Mullineaux P.M. (2008). Responses of the pea (*Pisum sativum* L.) leaf metabolome to drought stress assessed by nuclear magnetic resonance spectroscopy. Metabolomics.

[52] Bobille H., Fustec J., Robins R.J., Cukier C., Limami A.M. (2019). Effect of water availability on changes in root amino acids and associated rhizosphere on root exudation of amino acids in *Pisum sativum* L.. Phytochemestry.

[53] Du Y., Zhao Q., Chen L., Yao X., Xie F. (2020). Effect of drought stress at reproductive stages on growth and nitrogen metabolism in soybean. Agronomy.

[54] Yadav A.K., Carroll A.J., Estavillo G.M., Rebetzke G.J., Pogson B.J. (2019). Wheat drought tolerance in the field is predicted by amino acid responses to glasshouse-imposed drought. J. Exp. Bot..

[55] Michaletti A., Naghavi M.R., Toorchi M., Zolla L., Rinalducci S. (2018). Metabolomics and proteomics reveal drought-stress responses of leaf tissues from spring-wheat. Sci. Rep..

[56] Urano K., Maruyama K., Ogata Y., Morishita Y., Takeda M., Sakurai N., Suzuki H., Saito K., Shibata D., Kobayashi M. (2009). Characterization of the ABA-regulated global responses to dehydration in *Arabidopsis* by metabolomics. Plant J..

[57] Lee B.-R., Jin Y.L., Avice J.-C., Cliquer J.-B., Ourry A., Kim T.-H. (2009). Increased proline loading to phloem and its effects on nitrogen uptake and assimilation in water-stressed white clover (*Trifolium repens*). New Phytol..

[58] Simon-Sarkadia L., Kocsy G., Várhegyi A., Galiba G., de Ronde J.A. (2006). Effect of drought stress at supra-optimal temperature on polyamine concentrations in transgenic soybean with increased proline levels. Zeit. Nat. C.

[59] Meena M., Divyanshu K., Kumar S., Swapnil P., Zehra A., Shukla V., Yadav M., Upadhyay R.S. (2019). Regulation of L-proline biosynthesis, signal transduction, transport, accumulation and its vital role in plants during variable environmental conditions. Heliyon.

[60] Dar M.I., Naikoo M.I., Rehman F., Naushin F., Khan F.A., Iqbal N., Nazar R., Khan N.A. (2016). Proline accumulation in plants: Roles in stress tolerance and plant development. Osmolytes and Plants Acclimation to Changing Environment: Emerging Omics Technologies.

[61] Sharma S., Villamor J.G., Verslues P.E. (2011). Essential role of tissue-specific proline synthesis and catabolism in growth and redox balance at low water potential. Plant Physiol..

[62] Al-Quraan N.A., Al-Ajlouni Z.I., Qawasma N.F. (2021). Physiological and biochemical characterization of the GABA shunt pathway in pea (*Pisum sativum* L.) seedlings under drought stress. Horticulturae.

[63] Michaeli S., Fromm H. (2015). Closing the loop on the GABA shunt in plants: Are GABA metabolism and signaling entwined?. Front. Plant Sci..

[64] Fàbregas N., Fernie A.R. (2019). The metabolic response to drought. J. Exp. Bot..

[65] Li Z., Yuc J., Penga Y., Huang B. (2017). Metabolic pathways regulated by abscisic acid, salicylic acid and γ-aminobutyric acid in association with improved drought tolerance in creeping bentgrass (*Agrostis stolonifera*). Physiol. Plant..

[66] Tarkowski Ł.P., Signorelli S., Höfte M. (2020). γ-Aminobutyric acid and related amino acids in plant immune responses: Emerging mechanisms of action. Plant Cell Environ..

[67] Nakabayashi R., Mori T., Saito K. (2014). Alternation of flavonoid accumulation under drought stress in *Arabidopsis thaliana*. Plant Signal. Behav..

[68] Ros R., Muñoz-Bertomeu J., Krueger S. (2014). Serine in plants: Biosynthesis, metabolism, and functions. Trends Plant Sci..

[69] Wingler A., Quick W.P., Bungardi R.A., Bailey K.J., Lea P.J., Leegood R.C. (1999). The role of photorespiration during drought stress: An analysis utilizing barley mutants with reduced activities of photorespiratory enzymes. Plant Cell Environ..

[70] Blicharz S., Beemster G.T.S., Ragni L., De Diego N., Spìchal L., Hernándiz A.E., Marczak Ł., Olszak M., Perlikowski D., Kosmala A. (2021). Phloem exudate metabolic content reflects the response to water-deficit stress in pea plants (*Pisum sativum* L.). Plant J..

[71] Lahuta L.B., Pluskota W.E., Stelmaszewska J., Szablińska J. (2014). Dehydration induces expression of galactinol synthase and raffinose synthase in seedlings of pea (*Pisum sativum* L.). J. Plant Physiol..

[72] Pluskota W.E., Szablińska J., Obendorf R.L., Górecki R.J., Lahuta L. (2015). Osmotic stress induces genes, enzymes, and accumulation of galactinol, raffinose and stachyose in seedlings of pea (*Pisum sativum* L.). Acta Physiol. Plant..

[73] Nishizawa A., Yabuta Y., Shigeoka S. (2008). Galactinol and raffinose constitute a novel function to protect plants from oxidative damage. Plant Physiol..

[74] Lorence A., Chevone B.I., Mendes P., Nessler C.L. (2004). *Myo*-inositol oxygenase offers a possible entry point into plant ascorbate biosynthesis. Plant Physiol..

[75] Płonka J., Szablińska-Piernik J., Buszewski B., Baranowska I., Lahuta L.B. (2022). Analyses of antioxidative properties of selected cyclitols and their mixtures with flavanones and glutathione. Molecules.

[76] Yildizli A., Çevik S., Ünyayar S. (2018). Effects of exogenous *myo*-inositol on leaf water status and oxidative stress of *Capsicum annuum* under drought stress. Acta Physiol. Plant..

[77] ElSayed A.I., Rafudeen M.S., Golldack D. (2014). Physiological aspects of raffinose family oligosaccharides in plants: Protection against abiotic stress. Plant Biol..

[78] Iftime D., Hannah M.A., Peterbauer T., Heyer A.G. (2011). Stachyose in the cytosol does not influence freezing tolerance of transgenic Arabidopsis expressing stachyose synthase from adzuki bean. Plant Sci..

[79] Yang L., Fountain J.C., Ji P., Ni X., Chen S., Lee R.D., Kemerait R.C., Guo B. (2018). Deciphering drought-induced metabolic responses and regulation in developing maize kernels. Plant Biotech. J..

[80] Zhao M., Ren Y., Wei W., Yang J., Zhong Q., Li Z. (2021). Metabolite analysis of Jerusalem artichoke (*Helianthus tuberosus* L.) seedlings in response to polyethylene glycol-simulated drought stress. Int. J. Mol. Sci..

[81] Ding Y., Fromm M., Avramova Z. (2012). Multiple exposures to drought ‘train’ transcriptional responses in *Arabidopsis*. Nat. Commun..

[82] Couchoud M., Salon C., Girodet S., Jeudy C., Vernoud V., Prudent M. (2020). Pea efficiency of post-drought recovery relies on the strategy to fine-tune nitrogen nutrition. Front. Plant Sci..

[83] Jacques C., Salon C., Barnard R.L., Vernoud V., Prudent M. (2021). Drought stress memory at the plant cycle level: A review. Plants.

[84] Lisec J., Schauer N., Kopka J., Willmitzer L., Fernie A.R. (2006). Gas chromatography mass spectrometry-based metabolite profiling in plants. Nat. Protoc..

[85] Sun X., Weckwerth W. (2012). COVAIN: A toolbox for uni- and multivariate statistics, time-series and correlation network analysis and inverse estimation of the differential Jacobian from metabolomics covariance data. Metabolomics.

